# Retrolental Opacity: Atypical Manifestation of Lens-Induced Uveitis

**DOI:** 10.7759/cureus.93053

**Published:** 2025-09-23

**Authors:** Diego I Lopez-Zuñiga, Nestor Ibarra-Salazar, Sara Gonzalez-Godinez, Rodrigo Garza-Duron

**Affiliations:** 1 Institute of Ophthalmology and Visual Sciences, Tecnologico de Monterrey, Escuela de Medicina y Ciencias de la Salud, Monterrey, MEX

**Keywords:** anterior vitrectomy, hypermature cataract, intraocular lens, lens-induced uveitis, phacolytic uveitis, ultrabiomicroscopy

## Abstract

The crystalline lens can cause intraocular inflammation when its capsule is ruptured or allows leakage of lens proteins into the anterior chamber, a condition known as lens-induced uveitis (LIU). LIU is a rare form of uveitis and can be classified based on the integrity of the lens capsule. When the capsule is ruptured, the inflammation is called phacoantigenic or phacoanaphylactic uveitis, whereas if the capsule remains intact but leaks proteins, it is termed phacolytic uveitis. Common causes include ocular trauma, previous surgery, or spontaneous rupture in hypermature cataracts. Initial treatment often involves topical corticosteroids, but surgical intervention is frequently required.

We report the case of a 76-year-old male patient with a history of bilateral cataracts and prior cataract surgery in the left eye, who presented with decreased visual acuity and ocular pain in the right eye over several weeks. Examination revealed hand motion vision, normal intraocular pressure, fibrinous membranes in the anterior chamber, and a dense cataract with an intact anterior capsule. Ultrasound and ultrabiomicroscopy demonstrated punctiform echoes organized into a central plasmoid body adhered to the iris and corneal endothelium, in addition to a cataract with a dense and mobile central nucleus, absence of cortex, and an intact anterior capsule.

After medical management with topical steroids and nonsteroidal anti-inflammatory drugs to control inflammation, the patient underwent phacoemulsification with intraocular lens implantation. During surgery, a dense Morgagnian cataract with an intact capsule was removed. A whitish opacity behind the lens corresponding to anterior vitreous inflammation was identified and extracted via anterior vitrectomy. Postoperatively, inflammation improved significantly, and at two months, visual acuity recovered to 20/25 with complete resolution of ocular inflammation.

This case highlights the clinical presentation and management challenges of LIU, specifically phacolytic uveitis. The presence of a hypermature cataract in an eye without prior surgery or trauma strongly supports this diagnosis. While a phacoantigenic mechanism cannot be entirely excluded, the absence of prior lens capsule violation makes it highly unlikely. Recognizing these atypical inflammatory signs and implementing a combined medical and surgical approach are crucial to preserve vision and prevent complications.

## Introduction

The crystalline lens can be a source of intraocular inflammation when its capsule is ruptured or even when it remains apparently intact but allows proteins to leak into the anterior chamber. The term "lens-induced uveitis" (LIU) is used for this group of conditions and is often associated with hypermature or long-standing cataracts [1,2].

Although LIU is relatively rare, accounting for less than 1% of all uveitis cases, it represents an important cause of ocular morbidity and can pose diagnostic and therapeutic challenges, particularly in older adults or in regions with limited access to cataract surgery [2,3].

LIU can be classified into two main groups based on the integrity of the lens capsule. Phacoantigenic (or phacoanaphylactic) uveitis occurs when the lens capsule is ruptured, either spontaneously, such as in hypermature cataracts, or following trauma or surgery [4]. Phacolytic uveitis occurs when the capsule is microscopically intact but allows leakage of high-molecular-weight lens proteins, eliciting a non-granulomatous inflammatory reaction [2,4].

Clinical presentation of LIU can be variable, often including pain, redness, and decreased vision, which may complicate diagnosis due to overlap with other anterior segment inflammations. Causes of LIU include ocular trauma, previous surgery, or spontaneous rupture of the capsule in hypermature cataracts. Clinical identification of LUI is often challenging, and diagnosis is primarily made retrospectively [3,4]. Only six of 140 cases were diagnosed clinically in one review [5].

Since uveitis can manifest with varying degrees of severity, including an anterior chamber reaction with or without hypopyon, it is important to consider other potential causes based on the onset and clinical presentation in each patient. The differential diagnosis is broad and may include post-traumatic or postoperative endophthalmitis, sterile endophthalmitis, traumatic iritis, herpetic uveitis, anterior or intermediate uveitis, and Posner-Schlossman syndrome. Although sympathetic ophthalmia can resemble LIU, a key distinguishing feature is that in LIU the fellow eye remains unaffected, unlike in sympathetic ophthalmia [4].

Initial management typically involves topical and/or systemic corticosteroids, which can be effective in controlling inflammation; however, definitive surgical intervention, most commonly cataract extraction, is frequently required to resolve the inflammatory process and restore vision [1-3].

In this report, we present a case of phacolytic uveitis, highlighting the diagnostic challenges and the importance of timely surgical management, providing novel insights for clinicians encountering similar scenarios.

## Case presentation

A 76-year-old man, with no significant medical history, presented for consultation due to decreased visual acuity and ocular pain in the right eye for three weeks' duration. As ophthalmic antecedents, he reported a diagnosis of cataract in both eyes nine years prior and cataract surgery only in the left eye, as well as pterygium resection in the right eye five years ago, with no history of ocular trauma. 

**Figure 1:**
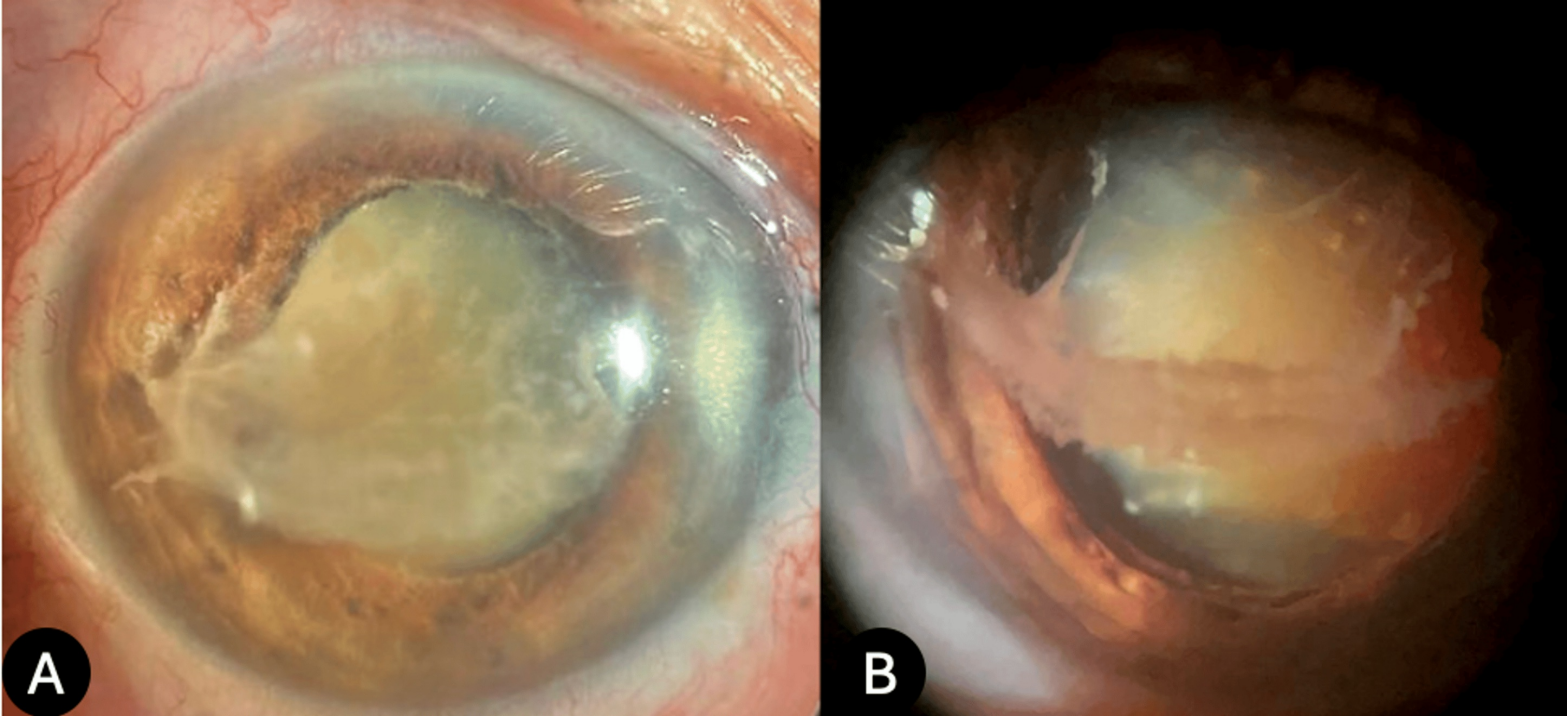
Slit lamp biomicroscopy of the right eye (A) Diffuse illumination and (B) lateral slit lamp view reveal a fibrinoid membrane on the central corneal endothelium.

Ophthalmological examination of the right eye revealed hand motion visual acuity that did not improve with pinhole, and there was an intraocular pressure of 15 mmHg, measured by Goldmann applanation tonometry. Biomicroscopy revealed a clear cornea without keratic precipitates. In the anterior chamber, whitish fibrinous membranes were observed extending from the iris border to the paracentral corneal endothelium, accompanied by the anterior chamber cell and flare (Figure 1).

The iris was without rubeosis or inflammatory nodules. A cataract with a dense, straw-yellow, crystallized-appearing nucleus was identified, which prevented visualization of the fundus, along with an absence of liquefied cortex material, as assessed by slit-lamp biomicroscopy.

Treatment was initiated with prednisolone acetate 1% ophthalmic suspension hourly and atropine sulfate 1% ophthalmic solution every eight hours, which led to a significant reduction in the plasmoid body and a decrease in pain and red eye.

**Figure 2:**
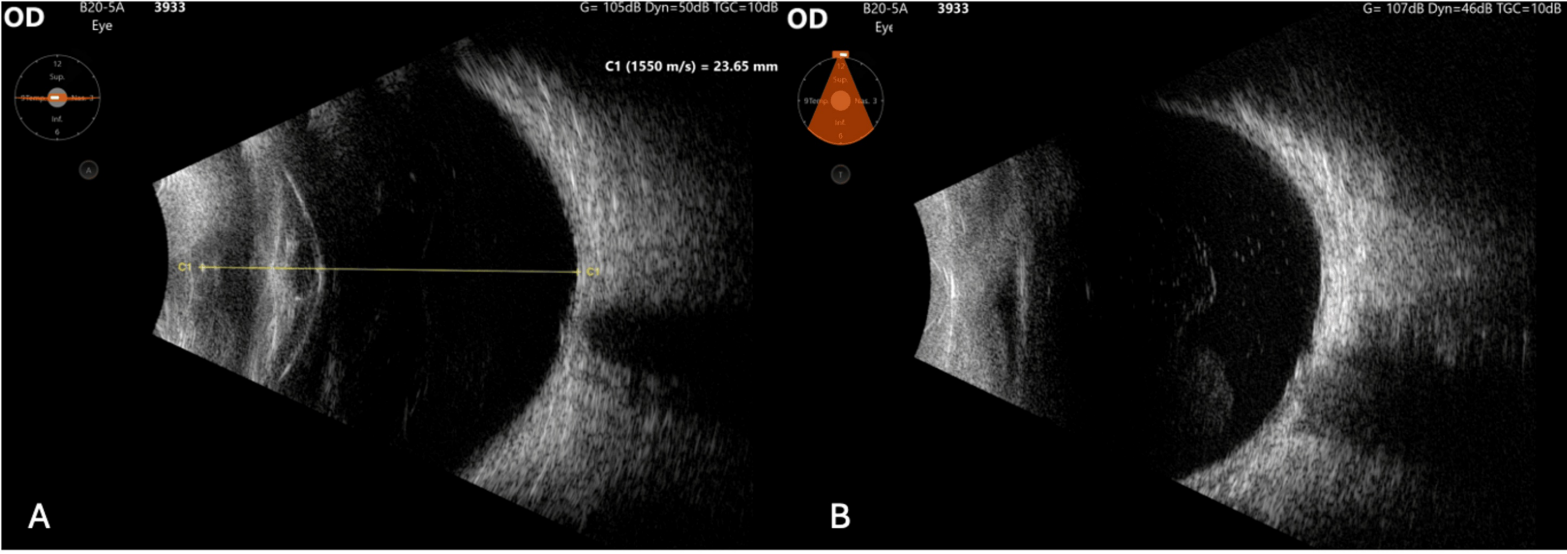
B-mode ultrasound of the right eye (A) Axial and (B) inferior transverse views, showing a dense cataract with fine and mobile punctiform echoes in the vitreous cavity

B-mode ultrasound was performed, demonstrating a dense cataract, fine mobile punctiform echoes within the anterior vitreous cavity, and a retina that remained attached (Figure 2).

**Figure 3:**
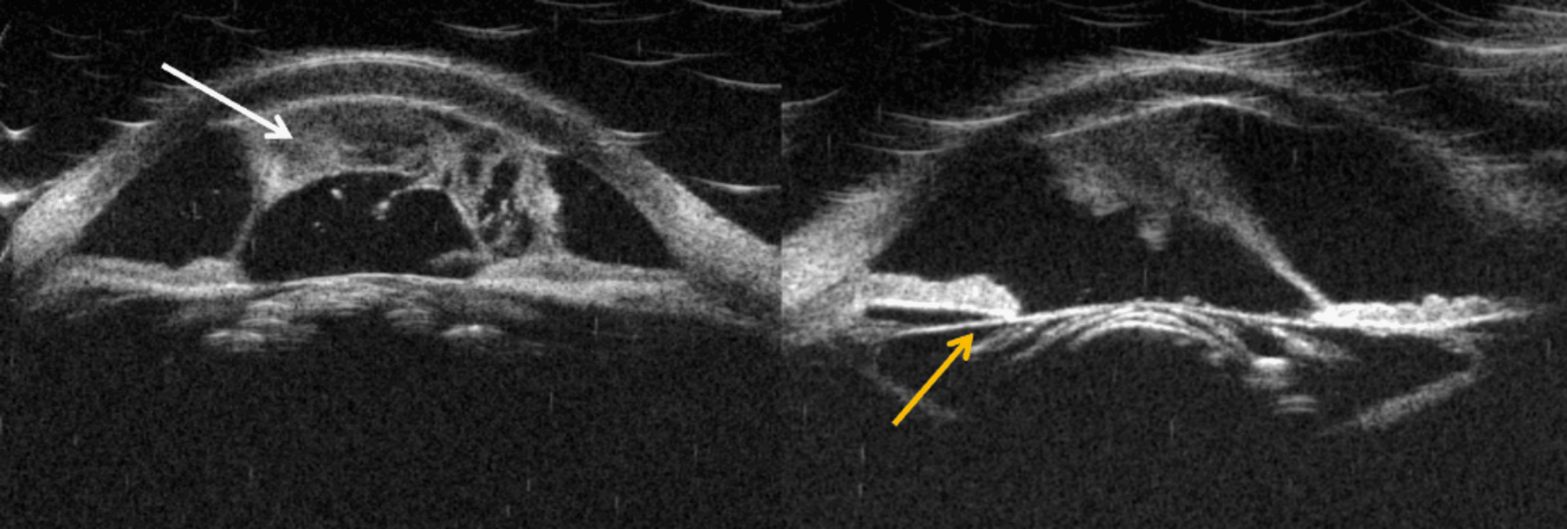
Ultrabiomicroscopy of the right eye Fibrinoid membrane adhered to the central corneal endothelium and iris (white arrow), in addition to a dense central nucleus with an intact anterior capsule (yellow arrow).

Ultrabiomicroscopy demonstrated abundant punctiform echoes organized into a central plasmoid body adhered to the iris and corneal endothelium, in addition to a cataract with a dense and mobile central nucleus, with absence of cortex and an intact anterior capsule (Figure 3). Given the clinical findings, a diagnosis of LIU was established. Surgical management with phacoemulsification and intraocular lens implantation was planned, following a uveitis protocol initiated three days before surgery to control inflammation. The treatment included prednisolone acetate 1% ophthalmic suspension every six hours, nepafenac 0.1% ophthalmic suspension every eight hours, oral prednisone 40 mg as a single dose and oral celecoxib 200 mg once daily.

**Figure 4:**
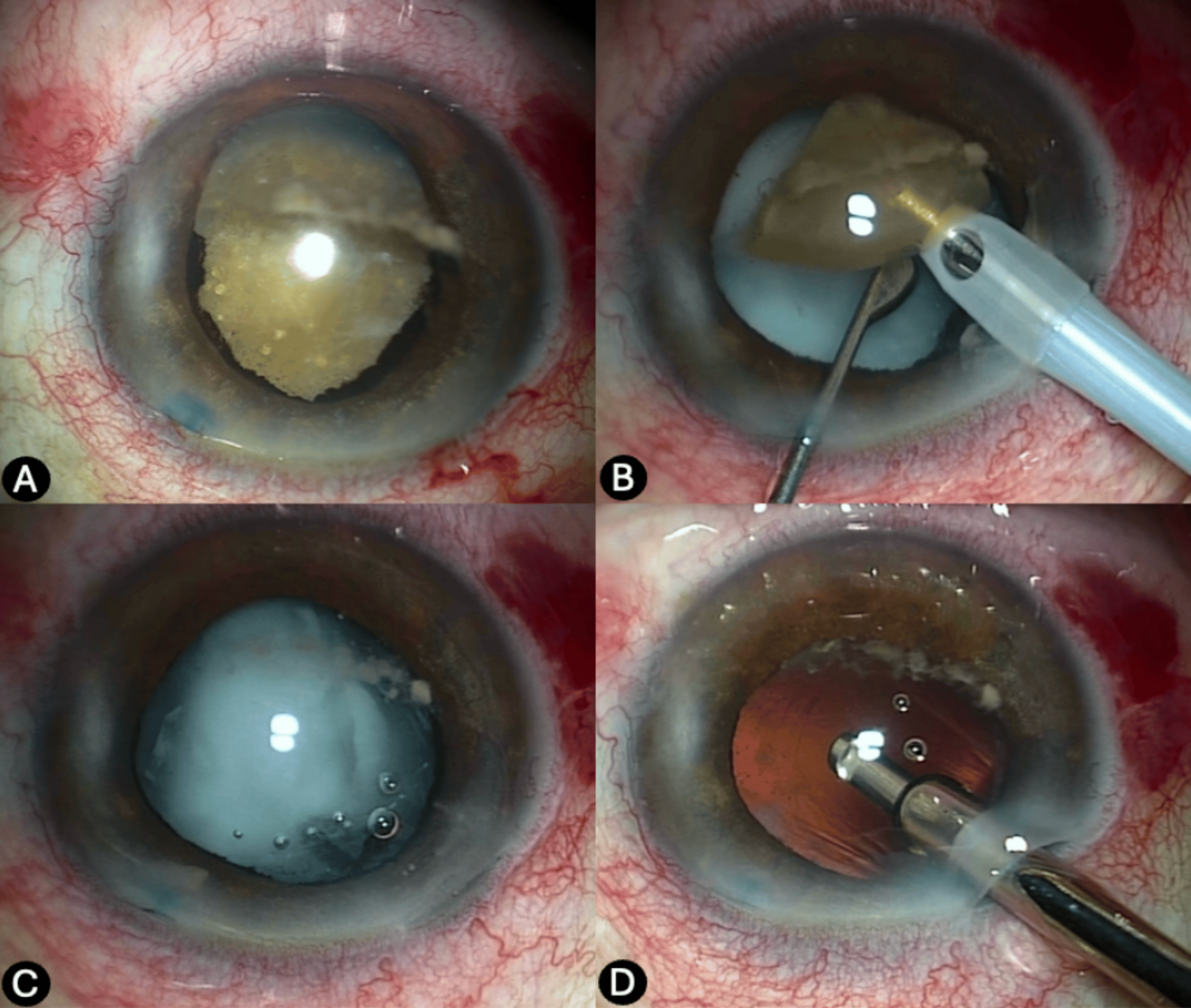
Clinical and surgical characteristics of the cataract (A) Preoperative appearance of the cataract, showing a straw-yellow, crystallized lens with an associated inflammatory fibrinoid membrane. (B) Phacoemulsification of the dense central nucleus. (C) Whitish, cotton-like retrolenticular inflammatory material. (D) Red reflex observed following removal of the retrolenticular material with anterior vitrectomy.

During surgery, a dense Morgagnian cataract with an intact anterior capsule was identified and successfully removed. After extraction of the lens material, a whitish, cotton-like, dense, and mobile retrolental opacity was observed, corresponding to an anterior vitreous opacity. Given this finding, an anterior vitrectomy was performed for its removal, and the intraocular lens was implanted in the sulcus (Figure 4).

In the immediate postoperative period, anterior chamber inflammation was graded as +3 cells according to the SUN classification, with associated stromal edema. Visual acuity improved to finger counting at 20 cm and intraocular pressure was 18 mmHg. Treatment with anti-inflammatory drugs and topical antibiotics was continued.

At two months, uncorrected visual acuity was 20/25, intraocular pressure was 13 mmHg and there was complete resolution of corneal and anterior segment inflammation.

## Discussion

LIU represents an infrequent but important form of intraocular inflammation, which can manifest with variable clinical signs depending on the integrity of the lens capsule and the magnitude of the host's immunological response. In the present case, the patient presented with a Morgagnian cataract with two weeks of ocular inflammation, with no recent history of trauma, which initially guided the differential diagnosis between phacolytic and phacoantigenic uveitis [1].

Phacoantigenic uveitis, also known as phacoanaphylactic uveitis, is mainly associated with a granulomatous immune reaction mediated by type IV delayed hypersensitivity, triggered by the exposure of lens proteins after a rupture of the lens capsule. Unlike phacolytic uveitis, which is usually non-granulomatous and secondary to an apparently intact capsule, phacoantigenic uveitis involves an evident or microscopic rupture of the capsule with sufficient antigenic exposure to cause severe inflammation, which can compromise vision if not treated promptly [2,3].

Although the classic inflammatory manifestations of LIU usually occur in the anterior chamber, characterized by cells, flare, and occasionally hypopyon, the presence of a whitish inflammatory material in the posterior chamber, as observed in this case, is less common but clinically significant. This appearance suggests more extensive inflammation, probably associated with the rupture or microperforation of the posterior capsule, allowing the release of lens proteins into the vitreous cavity and direct contact with the posterior capsule [1,4]. Previous studies and reports have documented that this posterior inflammation can generate visible deposits or exudates in the posterior chamber or anterior vitreous, complicating the picture and requiring more complex surgical interventions such as anterior vitrectomy in order to remove the inflammatory material and better control the immunological reaction [1].

The management of this condition requires a combined medical and surgical approach. Preoperative administration of non-steroidal anti-inflammatory drugs, along with topical and systemic corticosteroids, as applied in this case, has been shown to reduce the inflammatory component and facilitate surgery. The surgical intervention should focus on removing the antigenic source (the lens) and controlling any residual inflammation. The additional anterior vitrectomy was an appropriate measure due to the finding of persistent posterior inflammatory material. This strategy has been described in the literature as effective in preventing inflammatory recurrences and improving visual prognosis [1,4].

## Conclusions

LIU is an uncommon but potentially severe form of intraocular inflammation that may arise even without recent trauma or surgery. Its diagnosis requires a high index of suspicion, particularly in patients with hypermature cataracts who present with atypical inflammatory signs such as fibrinous anterior chamber reaction or anterior vitreous opacity. Over time, multiple confusing terms have been applied to this entity; currently, a simplified nomenclature based on lens capsule integrity, phacogenic versus phacolytic, is preferred. In the present case, the intact capsule and clinical features were consistent with phacolytic uveitis.

Timely management with intensive anti-inflammatory therapy followed by surgical lens extraction is essential to preserve vision and prevent complications. This case underscores the importance of considering LIU in the differential diagnosis of unilateral uveitis in elderly patients, especially when atypical presentations such as retrolental opacity are encountered.
